# A Novel Computation for Dimming Relative to the Adaptation Level in the Frog Retina

**DOI:** 10.1523/ENEURO.0061-26.2026

**Published:** 2026-09-11

**Authors:** Shun Murakami, Jun Umemoto, Miyu Horiguchi, Hiroshi Ishikane

**Affiliations:** ^1^Department of Psychology, Graduate School of Humanities, Senshu University, Kawasaki-shi, Kanagawa 214-8580, Japan; ^2^Department of Psychology, School of Human Sciences, Senshu University, Kawasaki-shi, Kanagawa 214-8580, Japan

**Keywords:** adaptation, coding, GABA, receptive field, retina, retinal ganglion cells

## Abstract

Ambient luminance varies widely, and retinal output is shaped by adaptation-dependent gain control. Relative to the adaptation level, a decrease in light intensity can either return intensity toward the adaptation level or drive it farther away from that level. Whether OFF retinal outputs signal both changes simply as light-intensity decrements or distinguish them according to their relationship to the adaptation level is currently unclear. This study examined OFF sustained retinal ganglion cells in bullfrogs (*Rana catesbeiana*) of either sex and quantified response bias by comparing spike counts during decrements that moved toward and away from the adaptation level. Responses fell into two functional populations: one signaled decrements in both cases, whereas the other selectively signaled decrements that moved away from the adaptation level, indicating adaptation level-referenced encoding. Although surround suppression was considered a possible contributor to these differences, manipulations expected to reduce classical surround contributions did not explain the selective suppression of responses during decrements that moved toward the adaptation level. Blocking GABA_A_ receptors (GABA_A_Rs) consistently weakened the response asymmetry between decrements that moved toward and away from the adaptation level, suggesting that GABA_A_R-dependent inhibition contributes to the mechanism underlying adaptation level-referenced responses. Taken together, these findings reveal a distinct computation for light-intensity decrement processing and expand the functional diversity of OFF outputs across light-intensity contexts.

## Significance Statement

This study reveals functional diversity among OFF sustained retinal ganglion cells (RGCs) in frogs in how they encode light-intensity decrements relative to the adaptation level. Rather than simply reporting decrements, cells fell into two functional groups: one responded to decrements that moved toward and away from the adaptation level, whereas the other selectively responded to decrements that moved away from the adaptation level. This distinction indicates that these OFF retinal outputs encode light-intensity decrements according to their relationship to the current visual context. These findings identify adaptation level-referenced responses in OFF sustained RGCs as a previously unrecognized form of OFF pathway processing.

## Introduction

Ambient luminance in natural environments spans many orders of magnitude, and the retina maintains sensitivity across this range through light adaptation. At low light intensity (scotopic), vision is mediated primarily by rods. Meanwhile, both rods and cones contribute at intermediate (mesopic) intensity, whereas cones dominate at high intensity (photopic; Stockman and Sharpe, 2006). Light adaptation is implemented both at the photoreceptor level and within downstream retinal circuits (Dunn et al., 2007; Ichinose and Lukasiewicz, 2007). For example, mean light intensity modulates synaptic gain in bipolar pathways (Ichinose and Lukasiewicz, 2007), and Dunn et al. (2007) showed that adaptation can emerge during signal transfer from cone bipolar cells to retinal ganglion cells (RGCs), thereby shaping the transmission of light increments and decrements to RGCs. More broadly, light adaptation entails gain regulation at multiple stages from photoreceptors to synaptic transmission in the inner retina.

RGCs are commonly classified into three types based on their light-evoked responses: ON RGCs respond to light increments, OFF RGCs respond to decrements, and ON–OFF RGCs respond to both (Hartline, 1938). Studies have demonstrated that these response properties depend on the adaptation level (Enroth-Cugell and Robson, 1966; Barlow and Levick, 1969; Enroth-Cugell and Lennie, 1975; Farrow et al., 2013; Pearson and Kerschensteiner, 2015; Tikidji-Hamburyan et al., 2015). For instance, surround suppression often changes with the adaptation level (Enroth-Cugell and Robson, 1966; Barlow and Levick, 1969; Enroth-Cugell and Lennie, 1975; Farrow et al., 2013), and in some cases, response polarity shifts with adaptation level (Pearson and Kerschensteiner, 2015; Tikidji-Hamburyan et al., 2015). In addition, studies in zebrafish have suggested that some OFF RGCs exhibit heterogeneous sensitivity profiles across light-intensity conditions (Robles et al., 2021).

However, previous research did not explicitly distinguish light-intensity changes by direction relative to the adaptation level. For light-intensity decrements, one direction is a decrement that moved toward the adaptation level, whereas the other is a decrement that moved away from that level. Even within the class of decrements, these two types of light-intensity changes might be processed differently in the retina. For example, in the natural environment of a frog, local dimming can occur due to the disappearance of water-surface reflections or bright patches produced by dappled sunlight; these dimming events may simply return light intensity to the adaptation level. In contrast, shadows cast by moving objects, such as predators, may produce decrements that drive light intensity away from the adaptation level. These cases highlight the need to clarify whether OFF retinal outputs encode decrements similarly across adaptation levels or show systematic differences depending on where the change occurs relative to the adaptation level. In this study, we examined whether responses to light-intensity decrements differ depending on their relationship to the adaptation level. Here, we define the adaptation level operationally as the reference light-intensity set by the preceding stimulus context (see Materials and Methods), against which subsequent decrements were classified as moving either toward or away from that level. To address this issue, we analyzed OFF sustained RGCs in the frog, which are known as dimming detectors (Lettvin et al., 1959; Grüsser and Grüsser-Cornehls, 1972). Because dimming detectors contribute to escape behavior in frogs (Ishikane et al., 2005), clarifying the dependence of their responses on light-intensity context might help elucidate the computations underlying dimming detection.

We quantified each cell's response bias using an index derived from spike counts evoked by light-intensity decrements that moved toward or away from the adaptation level and used this index to classify response profiles across cells. Using this approach, we identified two response patterns: one group of cells responded to decrements regardless of their direction relative to the adaptation level, whereas the other responded selectively to decrements that moved away from the adaptation level.

## Materials and Methods

### Retinal preparation and multielectrode array recording

In total, 53 frogs (*Rana catesbeiana*) of either sex were used (20 frogs for categorization of dimming detectors, seven frogs for adaptation-level and duration tests, nine frogs for surround suppression tests, and 17 frogs for pharmacological tests). Frogs were maintained under a 12 h light/dark cycle and dark-adapted for at least 60 min prior to experiments. All experimental procedures and animal handling protocols were approved by the Committee for Animal Experiments of Senshu University (approval nos. HP2024-1 and HP2025-1).

Multielectrode array (MEA) recordings were performed as described previously (Ishikane et al., 2005; Chaya et al., 2022; Umemoto et al., 2025). All surgical procedures were conducted under dim red light. After euthanasia, retinal pieces ∼3 mm in diameter were dissected and placed on an MEA (60pMEA200/30iR-Ti, Multi Channel Systems) with the ganglion cell layer facing down.

Extracellular spike activity was recorded and analyzed using Spike2 software (Cambridge Electronic Design) at a sampling rate of 20 kHz with a bandpass filter of 0.2–3 kHz. During recordings, each retina was continuously superfused with oxygenated Ringer's solution [100.0 mM NaCl, 2.5 mM KCl, 1.6 mM MgCl_2_, 1.0 CaCl_2_, 18.0 NaHCO_3_, and 10 glucose, supplemented with phenol red (4 mg/L)]. The solution was bubbled with a gas mixture of 95% O_2_ and 5% CO_2_. Recordings were performed at room temperature (∼23°C).

### Drugs

For pharmacological experiments, the following drugs were applied individually via bath perfusion at a final concentration of 10 µM: SR95531 (Sigma-Aldrich), TPMPA (Tocris Bioscience), and CGP55845 (Sigma-Aldrich), and 1 µM strychnine hydrochloride (Sigma-Aldrich). Stock solutions were prepared in distilled water and diluted 1,000-fold in Ringer's solution immediately before use.

### Visual stimulation

Visual stimuli were generated using GNU Octave with Psychtoolbox-3 (Pelli, 1997; Kleiner et al., 2007). Stimuli were displayed on a gamma-corrected organic light-emitting diode (OLED) monitor (refresh rate, 60 Hz; F13NA, Intehill) and projected onto the retina through an optical system. The light stimulus was presented over an area sufficiently larger than the retinal piece in the MEA chamber. Here, full-field stimulation refers to spatially uniform light stimulation of the entire retinal piece. The timing of stimulus onset was determined by recording the output of a photodiode positioned at the corner of the OLED monitor. Light intensity was measured with a calibrated spectrophotometer (FLAME-S, Ocean Optics) and converted to photoisomerization per rod per second (Rh*rod^−1^ s^−1^). The conversion was based on the absorption spectrum of frog rhodopsin (Donner et al., 1990) and rod dimensions adopted from a previous study (Bownds and Brodie, 1975), with correction for light absorption by phenol red in the Ringer’s solution.

**Figure 1.**
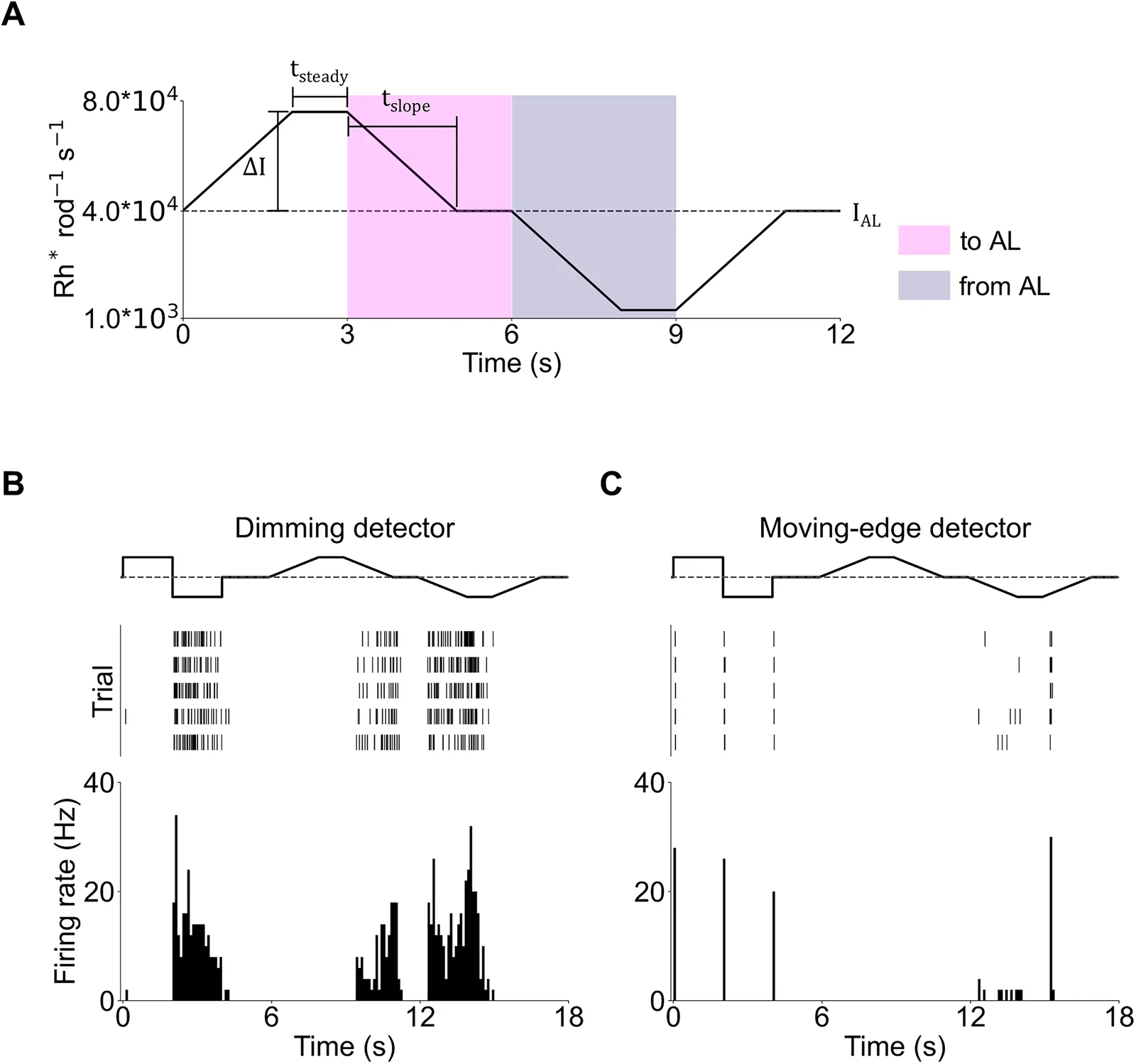
Slope stimulus and responses of the dimming and moving-edge detectors. ***A***, Time course of the slope stimulus. The stimulus was parameterized by the adaptation level (*I*_AL_), light-intensity change (Δ*I*), steady-phase duration (*t*_steady_), and slope phase duration (*t*_slope_). The magenta and blue-gray shaded regions indicate the to-AL and from-AL epochs, respectively, unless otherwise stated. ***B***, Peristimulus time histograms (PSTHs) for a dimming detector (left) and a moving-edge detector (right). Top, Stimulus light intensity. Middle, Spike rasters for repeated trials. Bottom, PSTH for each cell. PSTH bin width, 100 ms, unless otherwise stated.

Slope stimuli were used to identify dimming detectors and evaluate response bias between decrements that moved toward the adaptation level (to-AL) and away from the adaptation level (from-AL; Fig. 1*A*). The stimuli were defined by four parameters: adaptation level (*I*_AL_), Weber contrast (Δ*I*/*I*_AL_), steady-phase duration (*t*_steady_), and slope phase duration (*t*_slope_). Excluding the robustness across conditions experiment, in which either *I*_AL_ (8.0 × 10^4^ Rh*rod^−1^ s^−1^) or *t*_steady_ (4 s) was varied, the parameters were set to *I*_AL_ = 4.0 × 10^4^ Rh*rod^−1^ s^−1^, Δ*I*/*I*_AL_ = 0.90, *t*_steady_ = 1 s, and *t*_slope_ = 2 s. Based on previous measurements of frog rod photocurrents (Palacios et al., 1998), we treated the stimulus condition used in the present study as photopic throughout all stimulus phases. After adaptation of the retina to a light stimulus at *I*_AL_ for 5 min, the slope stimulus was presented six times. To minimize the influence of transient response changes at the onset of the stimulus sequence, the first trial was excluded from analysis. The results are thus based on the remaining five trials.

### Data analysis

Spikes were detected when the signal amplitude exceeded ±5 standard deviations of the baseline noise level (Babino et al., 2023; Umemoto et al., 2025). The baseline noise level was estimated from a 3 s period without visual stimulation. To exclude contaminated or poorly isolated units, only units exhibiting a clear refractory period in the autocorrelogram (bin width, 1 ms) were included in the analyses. Furthermore, duplicate recordings of the same neuron across multiple electrodes were identified by temporal cross-correlation, and one of the duplicate units was excluded (Li et al., 2014). Among the four major functional classes of frog RGCs, namely, sustained contrast detectors, net convexity detectors, moving-edge detectors, and dimming detectors, only moving-edge and dimming detectors respond to spatially uniform stimuli (Lettvin et al., 1959; Grüsser and Grüsser-Cornehls, 1972). In this study, dimming detectors, a class of OFF sustained RGCs, were analyzed. Dimming detectors exhibited sustained firing in response to light-intensity decrements (Fig. 1*B*, left), whereas moving-edge detectors exhibit transient firing in response to both light-intensity increments and decrements (Fig. 1*B*, right). Based on these properties, dimming detectors were identified as units that exhibited a firing rate exceeding 1 Hz during stimulus presentation and an ON–OFF index (OOI; Eq. 1) of less than −0.5. We defined *R*_ON_ as the total spike count during the increment epochs (*t* ∈ [0, 3) and [9, 12) s) and *R*_OFF_ as the total spike count during the decrement epoch (*t* ∈ [3, 9) s). Based on these variables, OOI was calculated as follows:

$$\mathrm{OOI}=\frac{R_{\mathrm{ON}}\;-\;R_{\mathrm{OFF}}}{R_{\mathrm{ON}\;}+\;R_{\mathrm{OFF}}}.$$

To avoid double-counting the same dimming detector across channels, only the dimming detector with the largest spike amplitude was selected for analysis. To evaluate response bias between dimming to and from AL, the adaptation level reference index (ALR index; Eq. 2) was calculated.

We defined *R*_to_ as the total spike count during the to-AL epoch (*t* ∈ [3, 6) s; [6, 12) s in the 4 s steady-phase condition) and *R*_from_ as the total spike count during the from-AL epoch (*t* ∈ [6, 9) s; [12, 18) s in the 4 s steady-phase condition):

$$\mathrm{ALR}\;\mathrm{index}=\frac{\;R_{\mathrm{from}}-R_{\mathrm{to}}}{\;R_{\mathrm{from}}+R_{\mathrm{to}}}.$$

We used a paired two-sided permutation test for statistical analyses. For comparisons between two conditions, the test statistic was defined as the median of the within-cell differences in the ALR index between the conditions. The null distribution was generated by randomly permuting the condition labels within each cell and repeating this procedure 1,000 times. As an effect size, we reported the median of differences between conditions. The *p* value was calculated as $p=(b+1)/(N_{\mathrm{perm}}+1)$, where b is the number of permutations yielding a test statistic with an absolute value greater than or equal to that observed $\left(|T_{\mathrm{perm}}|\geq |T_{\mathrm{obs}}|\right)$ and $N_{\mathrm{perm}}$ is the number of permutations.

## Results

### Dimming detectors can be categorized by adaptation level reference

**Figure 2.**
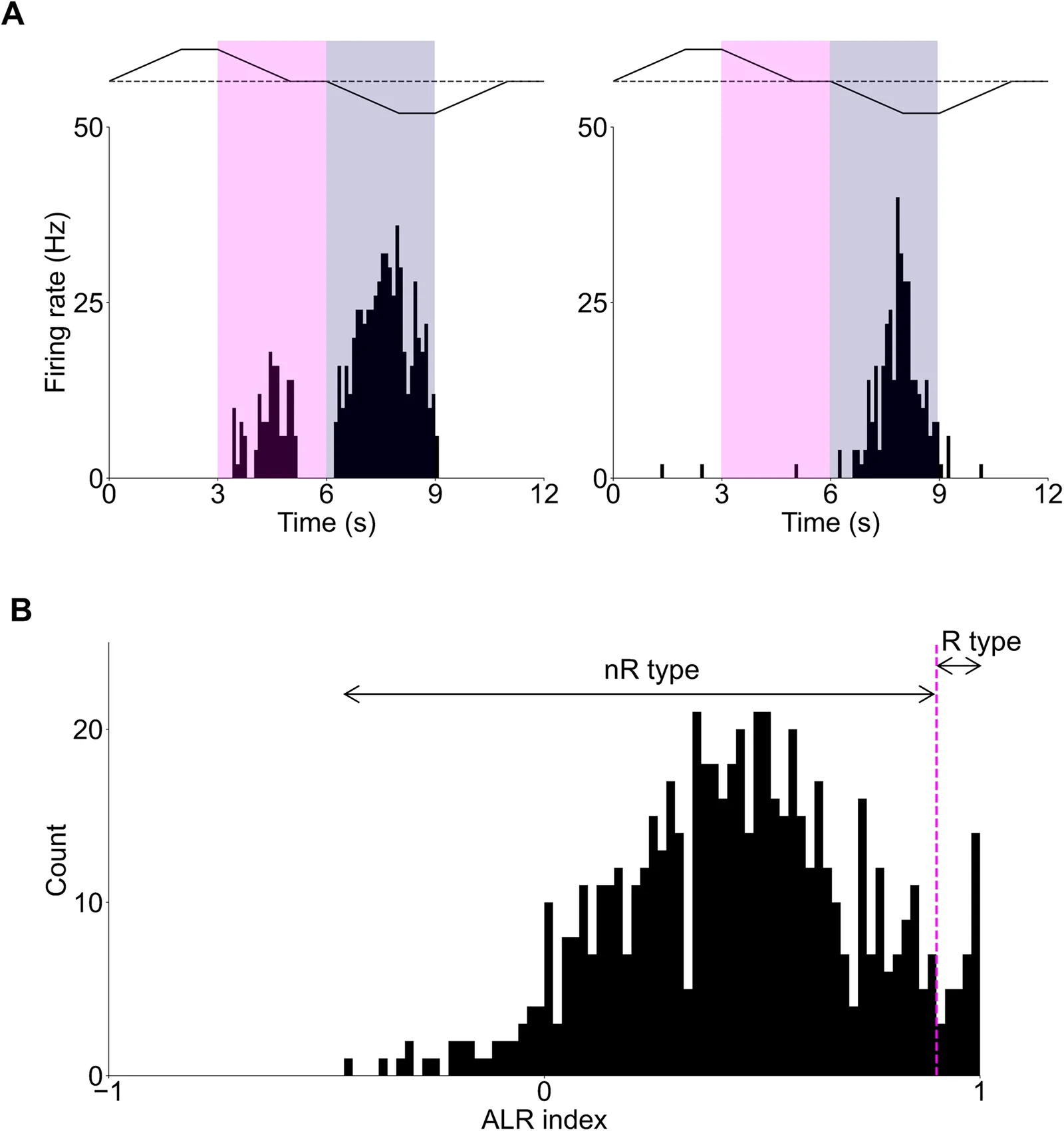
Peristimulus time histograms (PSTHs) of dimming detectors with distinct response profiles and distribution of the adaptation level reference index (ALR index). ***A***, Solid lines indicate light stimulus traces above the PSTHs and dashed lines indicate the adaptation level, unless otherwise stated. Left, A dimming detector responsive to decrements in both ranges of light intensity relative to the adaptation level (to-AL and from-AL; ALR index = 0.59). Right, A dimming detector that responds to light decrements only during the from-AL epoch (ALR index = 0.99). ***B***, Distribution of the ALR index across all cells (616 cells from 54 retinas and 20 frogs). The magenta vertical line (ALR index = 0.9) indicates the boundary used to categorize the dimming detectors into two groups. Mean PSTHs grouped by ALR index range are shown in Extended Data Figure 2-1.

Dimming detectors exhibited heterogeneous responses to the slope stimulus. Some cells responded to light-intensity decrements regardless of the direction relative to the adaptation level, resulting in a low ALR index (Fig. 2*A*, left). In contrast, other cells responded selectively during the from-AL epoch, yielding a high ALR index (Fig. 2*A*, right). The distribution of the ALR index showed a broad peak ∼0.4–0.5, with an additional group of cells concentrated near 1.0 (Fig. 2*B*). Few cells showed ALR indices ∼0.9 (Fig. 2*B*), and cells with ALR indices of 0.9–1.0 showed minimal response during the to-AL epoch (Extended Data Fig. 2-1). Based on this distribution and the response profiles during the to-AL epoch, cells were classified as follows: cells with an ALR index <0.9 were defined as adaptation level–nonreferenced cells (nR-type cells), whereas cells with an ALR index ≥0.9 were defined as adaptation level-referenced cells (R-type cells).

10.1523/ENEURO.0061-26.2026.f2-1 Figure 2-1 **Mean PSTHs for ALR index range from 0.5 to 1.0.** Orange: 0.5 ≤ALR index <0.6 (*n* = 86), yellow: 0.6 ≤ALR index <0.7 (*n* = 59), cyan: 0.7 ≤ALR index <0.8 (*n* = 45), blue: 0.8 ≤ALR index <0.9 (*n* = 38), magenta: 0.9 ≤ALR index <1.0 (*n* = 35). Data were obtained from 54 retinas of 20 frogs. Download Figure 2-1, TIF file.

### Adaptation level-referenced responses are consistently observed across the tested adaptation levels and steady-phase durations within the photopic range

**Figure 3.**
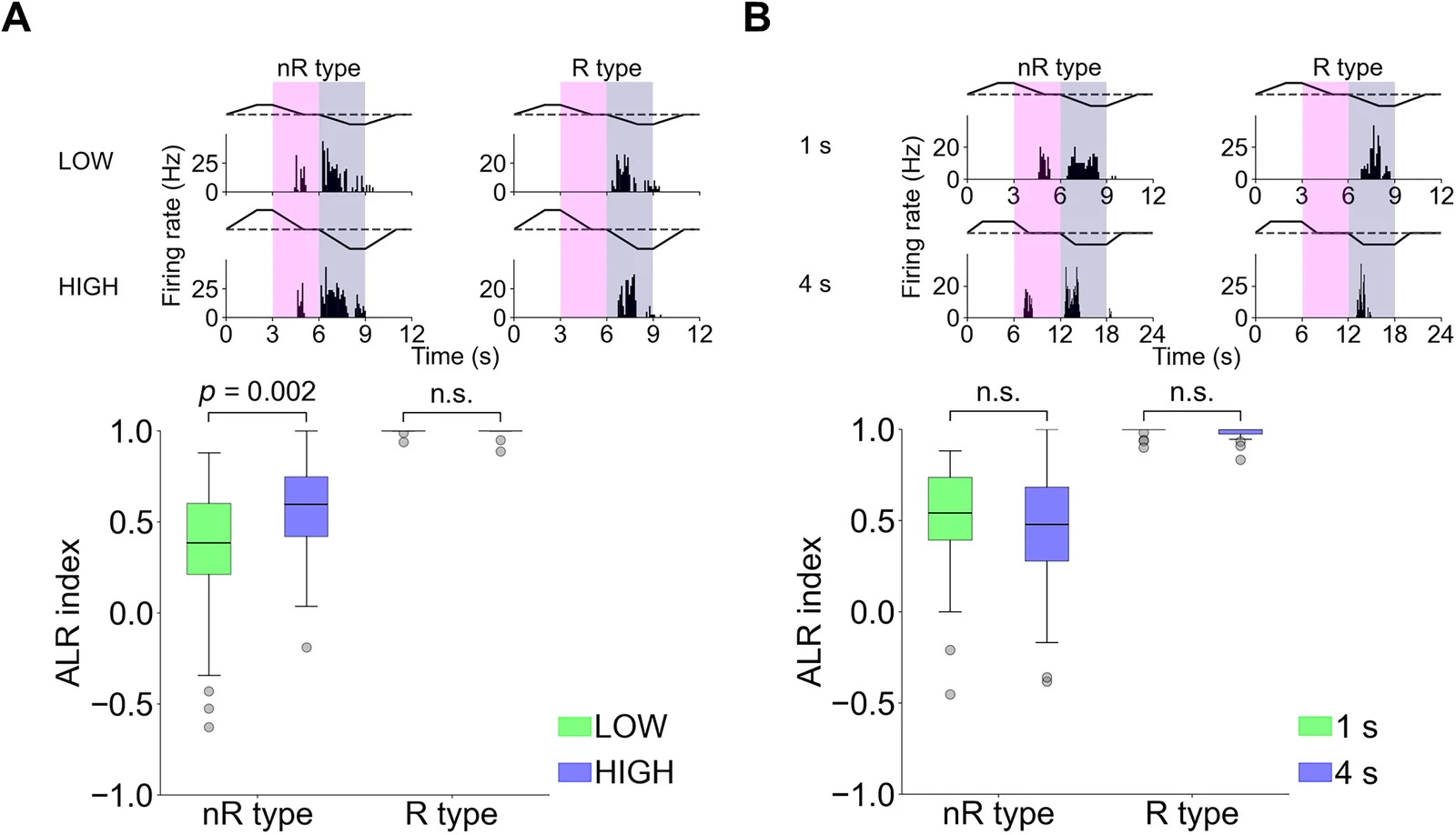
Representative peristimulus time histograms (PSTHs) and adaptation level reference index (ALR index) summaries across conditions. Representative PSTHs are presented for each experimental condition. LOW and HIGH responses, as well as 1 s and 4 s responses, were recorded from the same cells. The boxplots summarize the ALR index under each condition. The gray circle indicates an outlier (>IQR × 1.5). ***A***, Top, Representative PSTHs from nR-type (left) and R-type cells (right) at two adaptation levels (LOW; 4.0 × 10^4^ Rh*rod^−1^ s^−1^ and HIGH; 8.0 × 10^4^ Rh*rod^−1^ s^−1^). Bottom, Boxplots of the ALR index for nR-type (left) and R-type (right) cells at each adaptation level (green, LOW; blue, HIGH). nR-type: *n* = 87; R-type: *n* = 13 (nine retinas, three frogs). ***B***, Top, Representative PSTHs from nR-type (left) and R-type (right) cells at two steady-phase durations (1 and 4 s). Bottom, Boxplots of the ALR index for nR-type (left) and R-type (right) cells at each steady-phase duration (green, 1 s; blue, 4 s). nR-type: *n* = 94; R-type: *n* = 20 (12 retinas, four frogs).

These response characteristics might occur only at a specific adaptation level, as previous studies indicated that RGC responses vary with the adaptation level (Enroth-Cugell and Robson, 1966; Barlow and Levick, 1969; Enroth-Cugell and Lennie, 1975; Farrow et al., 2013; Pearson and Kerschensteiner, 2015; Tikidji-Hamburyan et al., 2015). To examine this possibility, responses were compared between two adaptation levels using stimuli with the same Weber contrast (0.90) across conditions (LOW: 4.0 × 10^4^ Rh*rod^−1^ s^−1^ and HIGH: 8.0 × 10^4^ Rh*rod^−1^ s^−1^). Cells were classified as nR- and R-type cells using the ALR index measured under the LOW condition. The ALR index of nR-type cells was significantly higher under the HIGH condition than under LOW condition (*n* *=* 87, median of differences = 0.169, *p* = 0.002; Fig. 3*A*). In contrast, the ALR index did not differ between the conditions for R-type cells (*n* *=* 13, median of differences = 0.000, *p* = 1.000; Fig. 3*A*). Analysis of spike counts revealed that in nR-type cells, spike counts at HIGH decreased during the to-AL epoch (*n* *=* 87, median of differences = −15, *p* = 0.002) but increased during the from-AL epoch (*n* *=* 87, median of differences = 31, *p* = 0.002). Thus, the increase in the ALR index observed for nR-type cells was accompanied by asymmetric changes in spike counts between the two epochs. In contrast, R-type cells did not exhibit significant changes in spike counts between the two adaptation levels, consistent with a stable ALR index (to-AL: *n* *=* 13, median of differences = 0, *p* = 1.000; from-AL: *n* *=* 13, median of differences = 20, *p* = 0.174).

Another possible explanation is that steady-phase adaptation is slower in R-type cells than in nR-type cells following a change in light intensity. Freeman et al. (2010) reported that luminance-dependent gain control in RGCs can be engaged within a few hundred milliseconds. To test whether differences in the time course of adaptation to the steady-phase light intensity could explain our results, responses were compared between two values of *t*_steady_ (1 and 4 s). Under *t*_steady_ = 1 s, neither nR-type cells nor R-type cells showed a significant change in the ALR index (nR-type: *n* *=* 94, median of differences = −0.032, *p* = 0.066; R-type: *n* *=* 20, median of differences = 0.000, *p* = 1.000; Fig. 3*B*). However, spike counts increased during both the to-AL and from-AL epochs under the 4 s duration in nR-type cells (to-AL: *n* *=* 94 cells, median of differences = 7.5, *p* = 0.002; from-AL: *n* *=* 94, median of differences = 16, *p* = 0.002). In R-type cells, spike counts during the from-AL epoch also increased under the 4 s duration (*n* *=* 20, median of differences = 15.5, *p* = 0.016), but no corresponding increase was observed during the to-AL epoch (*n* *=* 20, median of differences = 0, *p* = 1.000). In the 4 s condition, the increase in spike counts might reflect enhanced transient responses at the start and end of the slope phase. Overall, these results indicate that within the range tested, the differences in response characteristics between nR- and R-type cells do not arise solely from dependence on the specific adaptation level or from differences in the time required to adapt to the post-change steady-phase light intensity, but they are consistent with adaptation-level reference.

### Classical surround suppression is insufficient to account for adaptation level-referenced responses

Previous studies have reported that surround suppression is modulated by the adaptation level (Enroth-Cugell and Robson, 1966; Barlow and Levick, 1969; Enroth-Cugell and Lennie, 1975; Farrow et al., 2013). Accordingly, we tested whether adaptation level-referenced responses in dimming detectors could be explained by surround suppression by comparing responses to full-field and spot stimulation (diameter: 200, 400, and 800 μm). As the excitatory center of dimming detectors is estimated to span ∼1.2 mm (Tachibana, 2007), these spot stimuli are expected to primarily stimulate the excitatory center with minimal recruitment of the surround.

**Figure 4.**
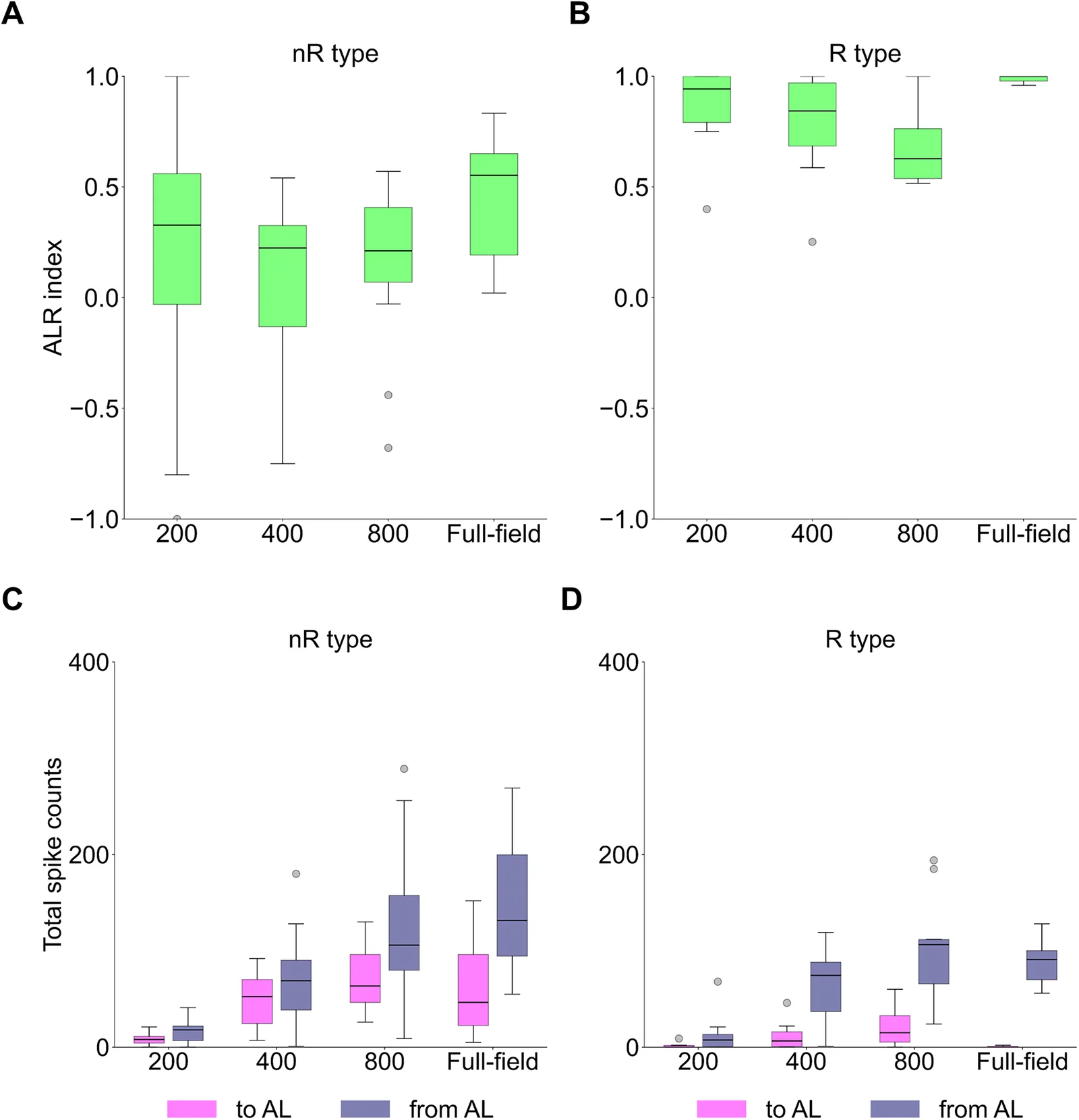
Adaptation level reference index (ALR index) and spike counts under each stimulus condition. Boxplots summarizing the ALR index and spike counts for nR-type and R-type cells. Spike counts are shown separately for each stimulus condition and epoch (magenta, to-AL epoch; blue-gray, from-AL epoch). The gray circle indicates an outlier. ***A***, ALR index for nR-type cells (*n* = 12). ***B***, ALR index for R-type cells (*n* = 10, with the exception of *n* = 7 for the 200 μm spot condition). ***C***, Spike counts for nR-type cells (*n* = 12). ***D***, Spike counts for R-type cells (*n* = 10). ***A*** and ***B*** were based on the cells available for each stimulus condition. Data were obtained from 10 retinas of five frogs. Additional ALR index and spike count analyses across stimulus sizes are shown in Extended Data Figure 4-1.

For nR-type cells, the ALR index did not differ significantly among the spot and full-field conditions (Fig. 4*A*, Table 1). Moreover, spike counts during both the to-AL and from-AL epochs generally increased with spot diameter, but the differences were not statistically significant (Fig. 4*C*, Table 2). Similarly, for R-type cells, the ALR index did not differ significantly among the spot and full-field conditions (Fig. 4*B*, Table 3), and spike counts during the from-AL epoch generally increased with spot diameter (Fig. 4*D*, Table 4). Notably, spike counts during the to-AL epoch changed little with increasing spot diameter, and the differences were not statistically significant (Fig. 4*D*, Table 4). Adaptation level-referenced responses are thus unlikely to be primarily explained by surround suppression. In an additional set of cells tested with larger spots, spike counts in nR- and R-type cells generally increased up to a spot diameter of ∼1.2 mm but showed little further increase at larger diameters (Extended Data Fig. 4-1*C,D*; Extended Data Tables 2-1, 4-1). The ALR index did not differ significantly among the spot and full-field conditions in either nR- or R-type cells (Extended Data Fig. 4-1*A,B*; Extended Data Tables 1-1, 3-1). These findings suggest that adaptation level-referenced responses cannot be explained by differences in the effective size of the excitatory center.

10.1523/ENEURO.0061-26.2026.f4-1 Figure 4-1 **Spike counts under each stimulus-size condition.** Boxplots summarizing the ALR index and spike counts for nR-type and R-type cells. Spike counts are shown separately for each stimulus condition and epoch (magenta: to-AL epoch, blue-gray: from-AL epoch). The gray circle indicates an outlier. ***A***, ALR index for nR-type cells (*n* = 11). ***B***, ALR index for R-type cells (*n* = 11). ***C***, Spike counts for nR-type cells (*n* = 11). ***D***, Spike counts for R-type cells (*n* = 11). A and B were based on the cells available for each stimulus condition. Data were obtained from nine retinas of four frogs. Download Figure 4-1, TIF file.

**Table 1.** Pairwise comparisons of the adaptation level reference index (ALR index) across stimulus conditions in nR-type cells

	200 µm	400 µm	800 µm	Full-field
200 µm		0.790 (−0.179)	0.790 (−0.187)	0.790 (0.187)
400 µm			0.790 (0.065)	0.085 (0.385)
800 µm				0.085 (0.345)
Full-field				

Values are Holm-adjusted *p* values; values in parentheses indicate median differences. Paired two-sided permutation tests were performed in nR-type cells (*n* = 12). Data were obtained from 10 retinas of five frogs. Additional statistical results for the ALR index at larger spot diameters are provided in Extended Data Table 1-1.

10.1523/ENEURO.0061-26.2026.t1-1 Table 1-1 **Pairwise comparisons of the ALR index across stimulus conditions in nR-type cells** Values are Holm-adjusted *p* values; values in parentheses indicate median differences. Paired two-sided permutation tests were performed in nR-type cells (*n* = 11). Data were obtained from nine retinas of four frogs. Download Table 1-1, DOCX file.

**Table 2.** Pairwise comparisons of spike counts across stimulus conditions in nR-type cells

To AL from AL	200 µm	400 µm	800 µm	Full-field
200 µm		0.090 (43.5)	0.090 (56.5)	0.090 (42.0)
400 µm	0.084 (57.5)		0.090 (17.0)	1.000 (0.5)
800 µm	0.084 (90.0)	0.084 (46.0)		0.476 (−23.0)
Full-field	0.084 (127.5)	0.084 (75.5)	0.563 (19.0)	

Values are Holm-adjusted *p* values; values in parentheses indicate median differences. Paired two-sided permutation tests were performed in nR-type cells (*n* = 12). The top and bottom triangles show comparisons for the to-AL and from-AL epochs, respectively. Data were obtained from 10 retinas of five frogs. Additional statistical results for spike counts at larger spot diameters are provided in Extended Data Table 2-1.

10.1523/ENEURO.0061-26.2026.t2-1 Table 2-1 **Pairwise comparisons of the ALR index across stimulus conditions in R-type cells** Values are Holm-adjusted *p* values; values in parentheses indicate median differences. Paired two-sided permutation tests were performed in R-type cells (*n* = 11). Data were obtained from nine retinas of four frogs. Download Table 2-1, DOCX file.

**Table 3.** Pairwise comparisons of the adaptation level reference index (ALR index) across stimulus conditions in R-type cells

	200 µm	400 µm	800 µm	Full-field
200 µm		0.501 (−0.148)	0.501 (−0.181)	0.501 (0.057)
400 µm			0.501 (−0.083)	0.181 (0.144)
800 µm				0.181 (0.372)
Full-field				

Values are Holm-adjusted *p* values; values in parentheses indicate median differences. Paired two-sided permutation tests were performed in R-type cells (*n* = 10, except for the 200 μm spot condition, for which *n* = 7). Data were obtained from 10 retinas of five frogs. Additional statistical results for the ALR index at larger spot diameters are provided in Extended Data Table 3-1.

10.1523/ENEURO.0061-26.2026.t3-1 Table 3-1 **Pairwise comparisons of spike counts across stimulus conditions in nR-type cells** Values are Holm-adjusted *p* values; values in parentheses indicate median differences. Paired two-sided permutation tests were performed in nR-type cells (*n* = 11). The upper and lower triangles show comparisons for the to-AL and from-AL epochs, respectively. Data were obtained from nine retinas of four frogs. Download Table 3-1, DOCX file.

**Table 4.** Pairwise comparisons of spike counts across stimulus conditions in R-type cells

To AL from AL	200 µm	400 µm	800 µm	Full-field
200 µm		0.202 (5.5)	0.202 (14.0)	1.000 (0.0)
400 µm	0.182 (59.5)		0.202 (7.5)	0.202 (−5.5)
800 µm	0.182 (86.5)	0.182 (21.0)		0.202 (−14.5)
Full-field	0.182 (79.0)	0.574 (24.0)	0.574 (−17.5)	

Values are Holm-adjusted *p* values; values in parentheses indicate median differences. Paired two-sided permutation tests were performed in R-type cells (*n* = 10). The top and bottom triangles show comparisons for the to-AL and from-AL epochs, respectively. Data were obtained from 10 retinas of five frogs. Additional statistical results for spike counts at larger spot diameters are provided in Extended Data Table 4-1.

10.1523/ENEURO.0061-26.2026.t4-1 Table 4-1 **Pairwise comparisons of spike counts across stimulus conditions in R-type cells** Values are Holm-adjusted *p* values; values in parentheses indicate median differences. Paired two-sided permutation tests were performed in R-type cells (*n* = 11). The upper and lower triangles show comparisons for the to-AL and from-AL epochs, respectively. Data were obtained from nine retinas of four frogs. Download Table 4-1, DOCX file.

### GABA_A_ receptors contribute to adaptation level-referenced responses

To identify inhibitory pathways that suppress dimming detectors, we tested receptor-specific antagonists targeting GABA_A_R (SR95531), GABA_C_R (TPMPA), glycineR (strychnine), and GABA_B_R (CGP55845). Drug effects were quantified by computing the ALR index for each cell and measuring spike counts during the to-AL and from-AL epochs.

**Figure 5.**
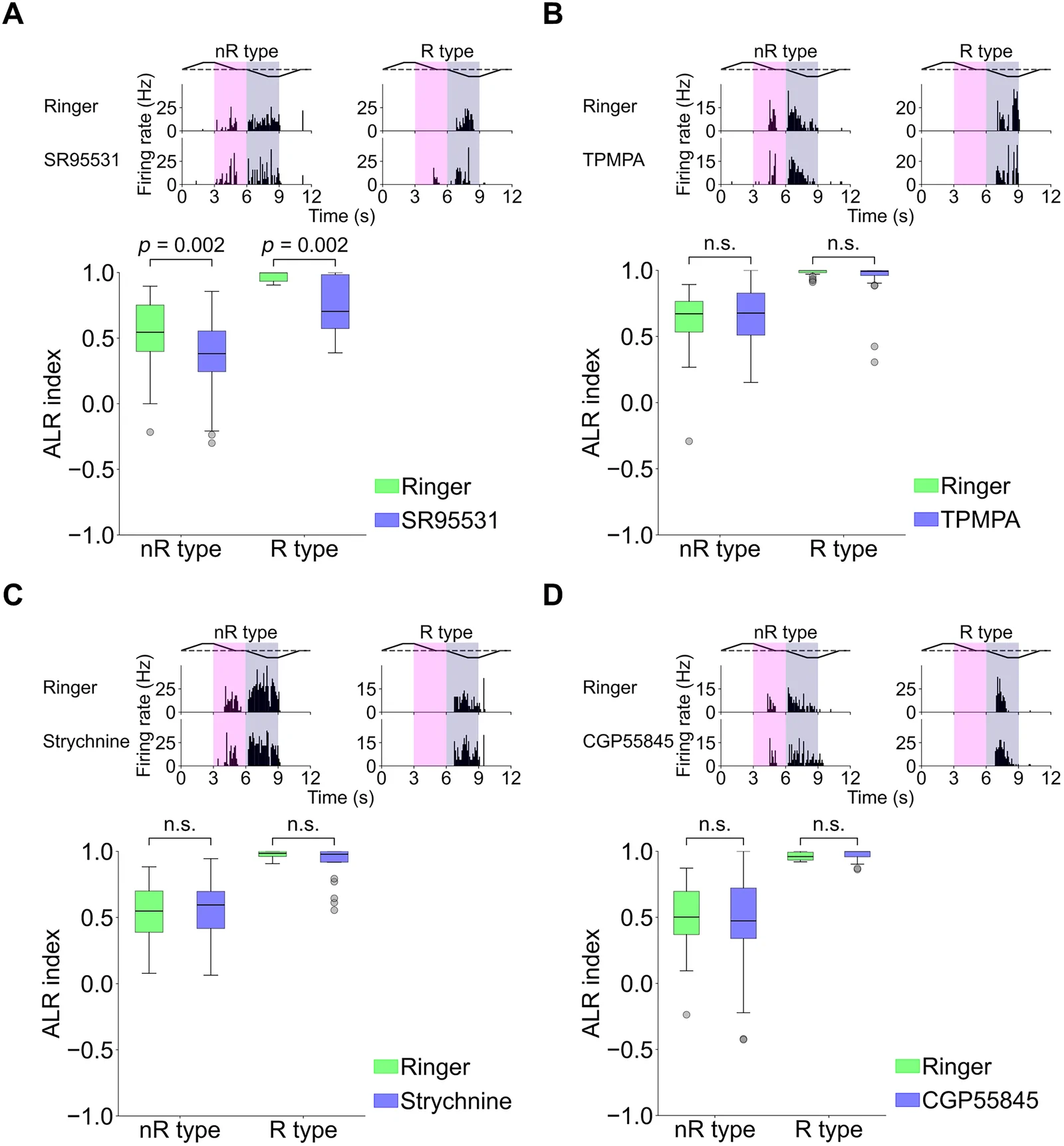
Representative peristimulus time histograms (PSTHs) and adaptation level reference index (ALR index) summaries of pharmacological experiments. Representative PSTHs are presented for Ringer and bath application of each drug. Ringer and drug responses were recorded from the same cells. Boxplots summarize the ALR index under each condition (green, Ringer; blue, drug). The gray circle indicates an outlier ***A***, SR95531. Top, Representative PSTHs from nR-type (left) and R-type (right) cells. Bottom, Boxplots of the ALR index for nR-type (left) and R-type (right) cells. nR-type: *n* = 60; R-type: *n* = 21 (12 retinas, four frogs). ***B***, TPMPA. Top, Representative PSTHs from nR-type (left) and R-type (right) cells. Bottom, Boxplots of the ALR index for nR-type (left) and R-type (right) cells. nR-type: *n* = 68; R-type: *n* = 30 (13 retinas, five frogs). ***C***, Strychnine. Top, Representative PSTHs from nR-type (left) and R-type (right) cells. Bottom, Boxplots of the ALR index for nR-type (left) and R-type (right) cells. nR-type: *n* = 63; R-type: *n* = 21 (12 retinas, four frogs). ***D***, CGP55845. Top, Representative PSTHs from nR-type (left) and R-type (right) cells. Bottom, Boxplots of the ALR index for nR-type (left) and R-type (right) cells. nR-type: *n* = 101; R-type: *n* = 16 (12 retinas, four frogs).

SR95531 decreased the ALR index in both nR- and R-type cells (nR-type: *n* *=* 60, median of differences = −0.159, *p* = 0.002; R-type: *n* *=* 21, median of differences = −0.247, *p* = 0.002; Fig. 5*A*). In nR-type cells, SR95531 selectively increased spike counts during the to-AL epoch (*n* *=* 60, median of differences = 15, *p* = 0.002) but not during the from-AL epoch (*n* *=* 60, median of differences = −5.5, *p* = 0.396). The same pattern was observed for R-type cells (to-AL: *n* *=* 21, median of differences = 19, *p* = 0.002; from-AL: *n* *=* 21, median of differences = 17, *p* = 0.543). These results indicate that GABA_A_R-mediated inhibition has an important role in adaptation level reference responses.

In contrast, TPMPA did not alter the ALR index in either cell type (nR-type, *n* *=* 68, median of differences = 0.041, *p* = 0.062; R-type: *n* *=* 30, median of differences = 0.000, *p* = 1.000; Fig. 5*B*), but it affected spike counts during the from-AL epoch (nR-type: *n* *=* 68, median of differences = 17.5, *p* = 0.006; R-type: *n* *=* 30, median of differences = 34, *p* = 0.032). Meanwhile, spike counts during the to-AL epoch remained unchanged (nR-type: *n* *=* 68, median of differences = −1, *p* = 0.681; R-type: *n* *=*30, median of differences = 0, *p* = 1.000). Thus, these results suggest that GABA_C_R-mediated inhibition modulates spiking during the from-AL epoch but does not specifically contribute to adaptation level-referenced responses, as quantified by the ALR index.

Strychnine did not affect the ALR index in either cell type (nR-type: *n* *=* 63, median of differences = 0.005, *p* = 0.913; R-type: *n* *=* 21, median of differences = 0.000, *p* = 1.000; Fig. 5*C*) or spike counts in either epoch (nR-type: to-AL, *n* *=* 63, median of differences = −6, *p* = 0.246; from-AL, *n* *=* 63, median of differences = 3, *p* = 0.817; R-type: to-AL, *n* *=* 21, median of differences = 0, *p* = 1.000; from-AL, *n* *=* 21, median of differences = −3, *p* = 0.947).

Similarly, CGP55845 did not affect the ALR index in either cell type (nR-type: *n* *=* 101, median of differences = −0.002, *p* = 0.863; R-type: *n* *=* 16, median of differences = 0.000, *p* = 1.000; Fig. 5*D*) or spike counts in either epoch (nR-type: to-AL, *n* *=* 101, median of differences = −3, *p* = 0.240; from-AL, *n* *=* 101, median of differences = −1, *p* = 0.873; R-type: to-AL, *n* *=* 16, median of differences = 0, *p* = 1.000; from-AL, *n* *=* 16, median of differences = −8.5, *p* = 0.547).

Overall, only GABA_A_Rs blockade consistently decreased the ALR index across nR- and R-type cells. In contrast, blocking GABA_C_Rs, glycineRs, or GABA_B_Rs did not measurably shift the ALR index despite changes in spike counts under some conditions.

## Discussion

Dimming detectors exhibited heterogeneous responses to the slope stimulus. We identified nR-type cells that responded to light-intensity decrements regardless of direction relative to the adaptation level and R-type cells that responded predominantly during the from-AL epoch with little responses during the to-AL epoch. This nR–R classification was preserved across the assessed adaptation levels and *t*_steady_ durations, and the adaptation level reference could not be explained solely by classical surround suppression. Blocking GABA_A_Rs increased responses during the to-AL epoch in both types, implicating GABA_A_R-mediated inhibition in establishing the adaptation level reference. These findings suggest that GABA_A_R-mediated inhibition enables adaptation level-referenced responses in a subset of dimming detectors, revealing functional diversity among OFF sustained RGCs and supporting the encoding of light-intensity decrements relative to the adaptation level.

### Consistency of adaptation level-referenced responses

nR-type cells exhibited an increase in the ALR index at the higher adaptation level, accompanied by an asymmetric change in spike counts between epochs (decreased to-AL and increased from-AL spike counts), suggesting that their responses are modulated by adaptation level. Such ALR index shifts in nR-type cells might partly reflect general adaptation-dependent changes in response magnitude, as reported previously (Enroth-Cugell and Robson, 1966; Barlow and Levick, 1969; Enroth-Cugell and Lennie, 1975; Farrow et al., 2013). Under this framework, adaptation-dependent changes in inhibitory balance could differentially affect the two epochs and thereby shift the ALR index in nR-type cells. In contrast, in R-type cells, the defining feature, namely, little spiking during the to-AL epoch, was maintained across adaptation levels, suggesting that adaptation level-referenced response characteristics were retained within this range. Because the light-intensity changes used in this experiment were slow, photoreceptors may have adapted to the steady-state light intensity reached during each epoch. However, photoreceptor adaptation alone cannot fully explain why R- and nR-type cells showed distinct response patterns under the same stimulus conditions. The preserved suppression of to-AL responses in R-type cells is thus unlikely to reflect only the instantaneous adaptation state of photoreceptors. These findings raise the possibility that retinal circuitry after the photoreceptors contributes to this reference-level dependence.

Although adaptation level-referenced responses were observed at both adaptation levels tested, the present analysis was limited to only two levels separated by 0.3 log units within a narrow photopic range. Moreover, the ALR index of R-type cells was already close to its upper limit, whereas that of nR-type cells increased at the higher adaptation level. Thus, whether adaptation level-referenced responses and the distinction between nR- and R-type cells are maintained across a broader range of adaptation levels remains to be determined.

### Limited contribution of surround suppression to adaptation level-referenced responses

Our spot stimulation experiments indicate that classical surround suppression makes only a limited contribution to adaptation level-referenced responses. If the response asymmetry were primarily generated by surround suppression, weakening the surround with spot stimulation would be expected to increase responses during the to-AL epoch, particularly in R-type cells. However, the ALR index was largely unchanged across full-field and spot conditions, and R-type cells maintained weak to-AL responses even when the spot diameter was varied. These results suggest that adaptation level-referenced responses cannot be explained solely by classical surround suppression.

The spot-size experiments also argue against a simpler explanation based on differences in the effective size of the excitatory center. Spike counts increased with spot diameter up to approximately the previously estimated center size of dimming detectors but showed little further increase at larger diameters. Thus, the difference between nR- and R-type cells is unlikely to reflect only differences in spatial summation within the excitatory center. Instead, adaptation level-referenced responses likely involve circuit mechanisms that selectively shape responses to light-intensity decrements depending on their direction relative to the adaptation level.

### GABAergic inhibition contributes to adaptation level-referenced responses

Among the antagonists tested, TPMPA increased spike counts, consistent with reduced GABA_C_R-dependent inhibition, yet it did not measurably change the ALR index. This dissociation suggests that GABA_C_R signaling primarily modulates response gain rather than the epoch asymmetry that defines adaptation level-referenced responses. GABA_C_Rs contribute broadly to retinal inhibition (Popova, 2015), and they can modulate presynaptic gain at bipolar cell terminals (Ichinose and Lukasiewicz, 2002; Hull et al., 2006; Oesch and Diamond, 2019). Thus, TPMPA increased firing without shifting the ALR index, supporting the role of GABA_C_Rs in gain control without specifically setting the adaptation level reference.

In contrast, GABA_A_Rs blockade increased spike counts and reduced the ALR index, indicating that GABA_A_R-mediated inhibition contributes to the asymmetry in spike counts across epochs that underlies adaptation level-referenced responses. Adaptation-related gain control has been proposed to occur in the inner plexiform layer (Dunn et al., 2007). Moreover, Farrow et al. (2013) suggested that GABAergic wide-field amacrine cells could contribute to adaptation-dependent changes in inhibition. Additionally, GABA_A_Rs are expressed in both bipolar and ganglion cells across retinas in vertebrates, including frogs (Brecha et al., 1991; Qian and Dowling, 1995; Du and Yang, 2000; Vitanova et al., 2001; Rotolo and Dacheux, 2003a,b). Thus, these observations suggest that adaptation level reference responses in dimming detectors are mediated, at least in part, by GABA_A_Rs within the inner plexiform layer, although a contribution from GABA_A_Rs in the outer plexiform layer cannot be excluded (Du and Yang, 2000; Vitanova et al., 2001). Mechanisms underlying RGC response diversity arise from differences in presynaptic inputs and intrinsic membrane properties. Swygart et al. (2024) reported that ganglion cell types with distinct response properties can share broadly similar intrinsic mechanisms, with functional diversification emerging from differences in presynaptic inputs. Meanwhile, the intrinsic membrane properties of RGCs shape spike output and contribute to response diversity (Milner and Do, 2017; Wienbar and Schwartz, 2022). Our observation that SR95531 increased spike counts in both nR- and R-type cells while shifting the ALR index downward is consistent with the idea that cell-to-cell differences in adaptation level-referenced responses reflect differences in inhibitory input rather than intrinsic differences in cellular mechanisms. Our results cannot be readily explained by intrinsic membrane properties alone: both nR- and R-type cells exhibited comparable OFF sustained responses during the from-AL epoch, whereas their responses diverged selectively during the to-AL epoch. This pattern suggests that adaptation level-referenced responses are more likely to arise from differences in presynaptic drive and/or inhibitory circuitry than from cell-intrinsic mechanisms alone.

Consistent with this circuit-based view, Matsumoto et al. (2025) reported extensive functional diversity among GABA-releasing amacrine cell types, including type-specific stratification, distinct release kinetics, and systematic offsets between receptive and projective fields. These properties provide a plausible substrate for heterogeneous inhibition. Accordingly, heterogeneous GABAergic amacrine cell inhibition might contribute to the diversity of adaptation level-referenced responses observed across dimming detectors by differentially shaping the inhibitory drive within the inner plexiform layer.

### Functional implications of adaptation level-referenced responses

One possible functional role of adaptation level-referenced responses to light-intensity decrements is to distinguish dimming events that potentially signal predator approach from nonthreatening changes in light intensity. As unnecessary movement can increase the risk that frogs are detected by predators (Nishiumi and Mori, 2015), this distinction is ecologically important. In natural settings, a looming shadow may indicate an approaching aerial predator and often produces from-AL dimming. In contrast, to-AL dimming caused by the disappearance of transient bright regions, such as water reflections or dappled sunlight, does not necessarily indicate predator approach. If to-AL dimming elicited escape behavior in all cases, the resulting movement may attract the attention of predators. From this perspective, R-type cells, which preferentially respond to from-AL dimming, could enhance the relative reliability of escape-relevant dimming signals while suppressing responses to less relevant dimming. This selectivity may therefore help reduce unnecessary responses to nonthreatening dimming events, although it may limit the range of light-intensity decrements represented by R-type cells.

In parallel, nR-type cells may provide a complementary channel that encodes light-intensity decrements across conditions. This parallel encoding scheme could allow the retina to emphasize escape-relevant from-AL dimming through R-type cells while preserving a broader representation of dimming signals through nR-type cells.

However, this functional interpretation should be considered with caution, because the slope stimuli used in the present study were simplified stimuli, and it remains unclear to what extent they reflect dimming events encountered in natural environments. Future studies using more naturalistic stimulus conditions will be necessary to evaluate the ecological relevance of adaptation level-referenced responses.

## Synthesis

Reviewing Editor: David Franklin, Technische Universitat Munchen Decisions are customarily a result of the Reviewing Editor and the peer reviewers coming together and discussing their recommendations until a consensus is reached. When revisions are invited, a fact-based synthesis statement explaining their decision and outlining what is needed to prepare a revision will be listed below. The following reviewer(s) agreed to reveal their identity: Christopher Passaglia. Note: If this manuscript was transferred from JNeurosci and a decision was made to accept the manuscript without peer review, a synthesis may not be available.

Two reviewers evaluated the manuscript "A novel computation for dimming relative to the adaptation level in the frog retina" and both reviewers agreed that the manuscript addresses an interesting question regarding luminance-dependent modulation of retinal ganglion cell responses, but also raised questions about the methods used, the analyses performed and the interpretations that may be made. In particular, the luminance regime, photoreceptor adaptation state, and functional identity of the recorded cells should be more rigorously characterized, and the interpretation involving surround mechanisms appears difficult to reconcile with the reported stimulus conditions. The have made detailed suggestions which you should find useful in revising your manuscript, if you decide to resubmit it to eNeuro or to another journal.

Major Issues

Methodological Concerns

1) What is the projected display area? Figure 4 gives impression it is barely larger than the ~1mm size of dimming detector receptive field centers, which would raise the possibility that the lower gain of R-type cells in Figure 4 might reflect those detectors having larger than normal center sizes. This would also mean the full-field stimulus partially fills their receptive field, which could explain their relative insensitivity to adaptation level compared to NR-type cells (since their adaptation pool would also be partially filled by comparison).

2) The manuscript does not clearly state the luminance regime under which the experiments were conducted (scotopic, mesopic, or photopic). This information is essential for interpreting the results. If the photoreceptors were operating in the Weber regime, the reported effects might indeed suggest additional circuit-level mechanisms. However, if the experiments were conducted outside the Weber regime, the observations could plausibly arise from incomplete photoreceptor adaptation or gain control at the level of rods or cones. It should be stated where 0.92 and 1.84 uW/cm2 fall within the luminosity range of frog RGCs. Presumably this is scotopic as methods indicate that frogs are dark-adapted for 1h before experiments and surgical procedures are performed under dim red light. How long was retina adapted to AL?

3) It is unclear whether phenol red was present in the recording medium and whether its spectral absorption was accounted for when estimating stimulus intensity. Because the interpretation of the experiments depends critically on the photoreceptor adaptation state, this issue should be clarified.

4) Which cells were recorded?

All recorded neurons are described simply as OFF ganglion cells. Given the substantial diversity of retinal ganglion cell types, this classification is insufficient to interpret the results. Numerous distinct OFF ganglion cell types are known even in relatively simple retinas, and functional differences across types could easily account for some of the variability reported here.

It would strengthen the manuscript substantially if the authors used independent functional measurements (e.g., receptive field size, temporal kinetics, mosaics, or other response features) to classify the recorded cells into putative types and then examined whether the reported luminance-dependent effects correlate with those types. Without this information, it is difficult to determine whether the findings represent a general property of OFF ganglion cells or simply reflect differences across cell classes.

Analysis Concerns

1) Based on Eq. 1, dimming detectors would have OOI > -0.5?

2) The bimodality of the distribution is highly questionable and the ALR cutoff is arbitrary. One could argue that the distribution is unimodal given the variability in counts and auto-clipping of ALR and that the "clear valley" begins at ALR of 0.7 instead of 0.9.

3) It is recommended that the impact of varying ALR cutoff index be explored, which would help justify the chosen one.

4) Box-and-whisker plots should be done for Figure 4 (like the other figures).

Interpretations concerns

1) The manuscript frequently describes responses as moving "toward" or "away from" the current adaptation level. This framing is potentially misleading given the slow luminance changes used in the experiments. Photoreceptor light adaptation occurs on relatively fast timescales, and the luminance changes used here appear modest (approximately a twofold change). Under such conditions, photoreceptors would likely have already adapted to the new luminance level by the time the stimulus reaches its steady state. Without quantitative reporting of stimulus intensity in photoreceptor-relevant units (e.g., photoisomerizations per photoreceptor per second), it is difficult to determine whether the reported effects are unexpected or consistent with known adaptation mechanisms.

2) Interpretation involving surround mechanisms

The interpretation that receptive field surround mechanisms contribute to the observed effects is difficult to reconcile with the stimulus configuration described in the manuscript. The authors note that the stimulus was smaller than the receptive field center of at least one of the ganglion cell types examined. If the stimulus does not extend beyond the center region, it is unclear how the surround could have been significantly engaged. This point raises a fundamental concern regarding the proposed mechanism.

L430-433. Statement is not supported by Figure 4 unless the stimulus size reduction is full to large spot; in which case, nR-type cells also showed the effect. Statement also seems to contradict L443-447.

3) Physiological relevance of the stimulus paradigm

The stimulus paradigm probes slow luminance changes over relatively long time scales. However, natural vision involves frequent and rapid changes in luminance at the level of individual receptive fields due to eye movements, head movements, and object motion. Because of this mismatch, it is unclear how the reported effects relate to visual processing under natural viewing conditions. A discussion of this limitation-or ideally experiments probing faster luminance transitions-would strengthen the physiological relevance of the work.

Specific comments

Abstract (lines 28-30): The sentence beginning with "However" is difficult to follow and should be clarified. The central finding should be understandable without reading the full manuscript.

Significance Statement (lines 50-51): Similar clarity issues arise here. The key result and its significance should be stated more directly.

Significance Statement (lines 55-56): The logic of the conclusion is not clear. The manuscript suggests that reduced dynamic range may be advantageous for detecting threats, but intuitively one would expect a larger dynamic range to improve sensitivity to salient stimuli. This reasoning should be clarified.

Paragraph beginning at line 92: The argument presented here would benefit from a concrete example illustrating when the proposed mechanism would operate during natural vision.

## References

[B1] Babino D, Benster TS, Laprell L, Van Gelder RN (2023) Assessment of murine retinal acuity ex vivo using multielectrode array recordings. Transl Vis Sci Technol 12:1–14. 10.1167/tvst.12.1.4 PMC9832724 36598460

[B2] Barlow HB, Levick WR (1969) Changes in the maintained discharge with adaptation level in the cat retina. J Physiol 202:699–718. 10.1113/jphysiol.1969.sp008836 5789945 PMC1351438

[B3] Bownds D, Brodie AE (1975) Light-sensitive swelling of isolated frog rod outer segments as an in vitro assay for visual transduction and dark adaptation. J Gen Physiol 66:407–425. 10.1085/jgp.66.4.407 52687 PMC2226212

[B4] Brecha NC, Sternini C, Humphrey MF (1991) Cellular distribution of L-glutamate decarboxylase (GAD) and gamma-aminobutyric acidA (GABAA) receptor mRNAs in the retina. Cell Mol Neurobiol 11:497–509. 10.1007/BF00734812 1660350 PMC11567406

[B5] Chaya T, Ishikane H, Varner LR, Sugita Y, Maeda Y, Tsutsumi R, Motooka D, Okuzaki D, Furukawa T (2022) Deficiency of the neurodevelopmental disorder-associated gene Cyfip2 alters the retinal ganglion cell properties and visual acuity. Hum Mol Genet 31:535–547. 10.1093/hmg/ddab268 34508581 PMC8863419

[B6] Donner K, Firsov ML, Govardovskii VI (1990) The frequency of isomerization-like “dark” events in rhodopsin and porphyropsin rods of the bull-frog retina. J Physiol 428:673–692. 10.1113/jphysiol.1990.sp018234 2231428 PMC1181669

[B7] Du JL, Yang XL (2000) Subcellular localization and complements of GABA(A) and GABA(C) receptors on bullfrog retinal bipolar cells. J Neurophysiol 84:666–676. 10.1152/jn.2000.84.2.666 10938294

[B8] Dunn FA, Lankheet MJ, Rieke F (2007) Light adaptation in cone vision involves switching between receptor and post-receptor sites. Nature 449:603–606. 10.1038/nature06150 17851533

[B9] Enroth-Cugell C, Lennie P (1975) The control of retinal ganglion cell discharge by receptive field surrounds. J Physiol 247:551–578. 10.1113/jphysiol.1975.sp010947 1142301 PMC1309488

[B10] Enroth-Cugell C, Robson JG (1966) The contrast sensitivity of retinal ganglion cells of the cat. J Physiol 187:517–552. 10.1113/jphysiol.1966.sp008107 16783910 PMC1395960

[B11] Farrow K, Teixeira M, Szikra T, Viney TJ, Balint K, Yonehara K, Roska B (2013) Ambient illumination toggles a neuronal circuit switch in the retina and visual perception at cone threshold. Neuron 78:325–338. 10.1016/j.neuron.2013.02.014 23541902

[B12] Freeman DK, Graña G, Passaglia CL (2010) Retinal ganglion cell adaptation to small luminance fluctuations. J Neurophysiol 104:704–712. 10.1152/jn.00767.2009 20538771 PMC4971895

[B13] Grüsser OJ, Grüsser-Cornehls U (1972) Comparative physiology of movement-detecting neuronal systems in lower vertebrates (Anura and Urodela). Bibli Ophthalmol 82:260–273. http://www.ncbi.nlm.nih.gov/pubmed/4631293 4631293

[B14] Hartline HK (1938) The response of single optic nerve fibers of the vertebrate eye to illumination of the retina. Am J Physiol 121:400–415. 10.1152/ajplegacy.1938.121.2.400

[B15] Hull C, Li G-L, von Gersdorff H (2006) GABA transporters regulate a standing GABAC receptor-mediated current at a retinal presynaptic terminal. J Neurosci 26:6979–6984. 10.1523/JNEUROSCI.1386-06.2006 16807327 PMC3572852

[B16] Ichinose T, Lukasiewicz PD (2002) GABA transporters regulate inhibition in the retina by limiting GABA(C) receptor activation. J Neurosci 22:3285–3292. 10.1523/JNEUROSCI.22-08-03285.2002 11943830 PMC6757522

[B17] Ichinose T, Lukasiewicz PD (2007) Ambient light regulates sodium channel activity to dynamically control retinal signaling. J Neurosci 27:4756–4764. 10.1523/JNEUROSCI.0183-07.2007 17460088 PMC3232015

[B18] Ishikane H, Gangi M, Honda S, Tachibana M (2005) Synchronized retinal oscillations encode essential information for escape behavior in frogs. Nat Neurosci 8:1087–1095. 10.1038/nn1497 15995702

[B19] Kleiner M, Brainard D, Pelli D (2007) What’s new in Psychtoolbox-3? Perception 36:14. 10.1177/03010066070360S101

[B20] Lettvin JY, Maturana HR, McCulloch WS, Pitts WH (1959) What the frog’s eye tells the frog’s brain. Proc IRE 47:1940–1951. 10.1109/JRPROC.1959.287207

[B21] Li PH, Field GD, Greschner M, Ahn D, Gunning DE, Mathieson K, Sher A, Litke AM, Chichilnisky EJ (2014) Retinal representation of the elementary visual signal. Neuron 81:130–139. 10.1016/j.neuron.2013.10.043 24411737 PMC3951785

[B22] Matsumoto A, Morris J, Looger LL, Yonehara K (2025) Functionally distinct GABAergic amacrine cell types regulate spatiotemporal encoding in the mouse retina. Nat Neurosci 28:1256–1267. 10.1038/s41593-025-01935-0 40234708 PMC12148929

[B23] Milner ES, Do MTH (2017) A population representation of absolute light intensity in the mammalian retina. Cell 171:865–876.e16. 10.1016/j.cell.2017.09.005 28965762 PMC6647834

[B24] Nishiumi N, Mori A (2015) Distance-dependent switching of anti-predator behavior of frogs from immobility to fleeing. J Ethol 33:117–124. 10.1007/s10164-014-0419-z

[B25] Oesch NW, Diamond JS (2019) Synaptic inhibition tunes contrast computation in the retina. Vis Neurosci 36:E006. 10.1017/S095252381900004X 31199207 PMC6578594

[B26] Palacios AG, Srivastava R, Goldsmith TH (1998) Spectral and polarization sensitivity of photocurrents of amphibian rods in the visible and ultraviolet. Vis Neurosci 15:319–331. 10.1017/s0952523898152136 9605532

[B27] Pearson JT, Kerschensteiner D (2015) Ambient illumination switches contrast preference of specific retinal processing streams. J Neurophysiol 114:540–550. 10.1152/jn.00360.2015 25995351 PMC4509391

[B28] Pelli DG (1997) The VideoToolbox software for visual psychophysics: transforming numbers into movies. Spat Vis 10:437–442. 10.1163/156856897X00366 9176953

[B29] Popova E (2015) GABAergic neurotransmission and retinal ganglion cell function. J Comp Physiol A Neuroethol Sens Neural Behav Physiol 201:261–283. 10.1007/s00359-015-0981-z 25656810

[B30] Qian H, Dowling JE (1995) GABAA and GABAC receptors on hybrid bass retinal bipolar cells. J Neurophysiol 74:1920–1928. 10.1152/jn.1995.74.5.1920 8592185

[B31] Robles E, Fields NP, Baier H (2021) The zebrafish visual system transmits dimming information via multiple segregated pathways. J Comp Neurol 529:539–552. 10.1002/cne.24964 32484919

[B32] Rotolo TC, Dacheux RF (2003a) Evidence for glycine, GABAA, and GABAB receptors on rabbit OFF-alpha ganglion cells. Vis Neurosci 20:285–296. 10.1017/s0952523803203072 14570250

[B33] Rotolo TC, Dacheux RF (2003b) Two neuropharmacological types of rabbit ON-alpha ganglion cells express GABAC receptors. Vis Neurosci 20:373–384. 10.1017/s095252380320403x 14658766

[B34] Stockman A, Sharpe LT (2006) Into the twilight zone: the complexities of mesopic vision and luminous efficiency. Ophthalmic Physiol Opt 26:225–239. 10.1111/j.1475-1313.2006.00325.x 16684149

[B35] Swygart D, Yu W-Q, Takeuchi S, Wong ROL, Schwartz GW (2024) A presynaptic source drives differing levels of surround suppression in two mouse retinal ganglion cell types. Nat Commun 15:599. 10.1038/s41467-024-44851-w 38238324 PMC10796971

[B36] Tachibana M (2007) Neuronal synchronous discharges and behavior: retinal synchronous discharges are essential for escape behavior in frogs. Seibutsu Butsuri 47:355–361. 10.2142/biophys.47.355

[B37] Tikidji-Hamburyan A, Reinhard K, Seitter H, Hovhannisyan A, Procyk CA, Allen AE, Schenk M, Lucas RJ, Münch TA (2015) Retinal output changes qualitatively with every change in ambient illuminance. Nat Neurosci 18:66–74. 10.1038/nn.3891 25485757 PMC4338531

[B38] Umemoto J, Murakami S, Ishikane H (2025) Long-range precise synchronous firing and receptive field encoding in the frog retina. J Neurophysiol 135:233–240. 10.1152/jn.00566.2025 41442146

[B39] Vitanova L, Kupenova P, Haverkamp S, Popova E, Mitova L, Wässle H (2001) Immunocytochemical and electrophysiological characterization of GABA receptors in the frog and turtle retina. Vision Res 41:691–704. 10.1016/s0042-6989(00)00294-7 11248259

[B40] Wienbar S, Schwartz GW (2022) Differences in spike generation instead of synaptic inputs determine the feature selectivity of two retinal cell types. Neuron 110:2110–2123.e4. 10.1016/j.neuron.2022.04.012 35508174 PMC9262831

