# Supplementary material for: A Novel Computation for Dimming Relative to the Adaptation Level in the Frog Retina
Source: eNeuro. 2026 Sep 11;13(9):ENEURO.0061-26.2026. doi: 10.1523/ENEURO.0061-26.2026 (PMC13576493; doi:10.1523/ENEURO.0061-26.2026)
Supplement: Supplementary file 2 [file eneuro-13-ENEURO.0061-26.2026-s005.docx]

|  | 800 µm | 1200 µm | 1600 µm | Full-field |
| --- | --- | --- | --- | --- |
| 800 µm |  | 0.189 (－0.146) | 0.189 (－0.231) | 0.571 (0.025) |
| 1200 µm |  |  | 1.000 (0.009) | 0.189 (0.260) |
| 1600 µm |  |  |  | 0.189  (0.321) |
| Full-field |  |  |  |  |

**Table 3-1. Pairwise comparisons of the ALR index across stimulus conditions in R-type cells**

Values are Holm-adjusted *p* values; values in parentheses indicate median differences. Paired two-sided permutation tests were performed in R-type cells (*n* = 11). Data were obtained from nine retinas of four frogs.
