# Supplementary material for: A Novel Computation for Dimming Relative to the Adaptation Level in the Frog Retina
Source: eNeuro. 2026 Sep 11;13(9):ENEURO.0061-26.2026. doi: 10.1523/ENEURO.0061-26.2026 (PMC13576493; doi:10.1523/ENEURO.0061-26.2026)
Supplement: Supplementary file 3 [file eneuro-13-ENEURO.0061-26.2026-s006.docx]

| to AL  from AL | 800 µm | 1200 µm | 1600 µm | Full-field |
| --- | --- | --- | --- | --- |
| 800 µm |  | 0.184 (38.0) | 0.500 (35.0) | 0.184 (65.0) |
| 1200 µm | 0.185 (22.0) |  | 0.757 (-3.0) | 0.500 (25.0) |
| 1600 µm | 0.185 (27.0) | 1.000 (3.0) |  | 0.587  (25.0) |
| Full-field | 1.000 (14.0) | 1.000 (－18.0) | 1.000 (－15.0) |  |

**Table 2-1. Pairwise comparisons of spike counts across stimulus conditions in nR-type cells**

Values are Holm-adjusted *p* values; values in parentheses indicate median differences. Paired two-sided permutation tests were performed in nR-type cells (*n* = 11). The upper and lower triangles show comparisons for the to-AL and from-AL epochs, respectively. Data were obtained from nine retinas of four frogs.
